# Knowledge and Barriers About Blood Donation and Associated Factors in Saudi Arabia: A Systematic Review

**DOI:** 10.7759/cureus.48506

**Published:** 2023-11-08

**Authors:** Afaf E Alanazi, Bader Ramadan F Almulla, Salman Meshal S Alanazi, Sagir Khalaf M Alshammari, Abdullah Ali A Aldossary, Saud Ghadeer M Alanazi, Rayan Abdulaziz S Alenezi, Turki Manwi B Alanazi

**Affiliations:** 1 Family Medicine, Ministry of Health, Arar, SAU; 2 General Medicine, Northern Border University, Arar, SAU; 3 Medicine, Northern Border University, Arar, SAU; 4 General Practice, Northern Border University, Arar, SAU; 5 General Medicine, Northen Border University, Arar, SAU; 6 General Medicine and Surgery, Northern Border University, Arar, SAU

**Keywords:** barriers, systematic review, saudi arabia, blood transfusion, blood donation

## Abstract

Transfusions and blood donations are relatively risk-free medical procedures. However, attitudes, beliefs, and knowledge levels about blood donation and transfusion may influence such operations. This systematic review aims to comprehensively investigate the level of knowledge and attitudes towards blood donation in Saudi Arabia. PubMed, Scopus, Web of Science, and ScienceDirect were systematically searched for relevant literature. Rayyan (Qatar Computing Research Institute, Ar-Rayyan, Qatar) was employed throughout this comprehensive process. This review included 17 studies with a total of 9,212 patients, and 4,806 (52.2%) were males.

The majority of Saudis lacked awareness about blood donation and had unfavorable opinions towards it. In addition to various fears, mistrust, a lack of information, and not being approached by anyone to donate blood, participants reported that the distance to the donation site, the difficulty with transportation, the time commitment, getting a quick break from work or the office, or taking time off from home were also contributing to the negative attitudes towards blood donation. While youthful participants were more likely to contribute, Saudi university students in this study had inadequate information but positive attitudes about blood donation. Ineligibility, fear of giving blood, and inaccurate information regarding blood donation are the main deterrents to blood donation.

## Introduction and background

Blood is a unique form of bodily fluid with unique components that transports various metabolic waste products and necessary elements like oxygen and nutrition to the body's cells [1]. Blood donation is the procedure of obtaining blood from willing donors who are unlikely to endanger their health and are at low risk for infection [2]. For those who experience blood loss due to car accidents, surgery, pregnancy problems, chemotherapy, and disorders including malaria, anemia, and intestinal parasites that increase the need for blood, it is a life-saving procedure [3].

The World Health Organization (WHO) promotes the voluntary and unpaid donation of blood [4]. According to the benevolence hypothesis, blood donors, as opposed to non-donors, have a general preference for altruistic motives based on a warm glow (i.e., "I donate because it makes me feel good"). The evidence is in favor of the benevolence theory, which implies that some blood donors have selfish motivations. Blood donation campaigns ought to emphasize charitable themes rather than only altruistic ones [5].

Blood donation is a crucial aspect of healthcare, and it is essential to have a sufficient supply of blood to meet the needs of patients in need. In Saudi Arabia, there is a growing concern regarding the knowledge and barriers associated with blood donation. It is important to understand the factors that influence blood donation behavior in Saudi Arabia to develop effective interventions that can increase the number of blood donors. One of the significant factors that influence blood donation behavior in Saudi Arabia is the lack of knowledge about the importance of blood donation. Many people in Saudi Arabia are unaware of the benefits of blood donation and how it can save lives. It is crucial to educate the public about the importance of blood donation and how it can contribute to the healthcare system in Saudi Arabia [6].

Another significant barrier to blood donation in Saudi Arabia is the fear of needles. Many people in Saudi Arabia are afraid of needles, which makes them hesitant to donate blood. It is essential to address this fear and provide reassurance to potential blood donors that the process is safe and painless. Moreover, cultural and religious beliefs can also act as barriers to blood donation in Saudi Arabia. Some people believe that blood donation is against their religious beliefs, while others are hesitant due to cultural norms and beliefs. It is important to address these concerns and provide culturally sensitive information to potential blood donors [6, 7]. In addition to these factors, there are also logistical barriers to blood donation in Saudi Arabia. The lack of blood donation centers and the limited availability of blood donation drives can make it difficult for people to donate blood. It is essential to increase the number of blood donation centers and organize more blood donation drives to make it easier for people to donate blood [6].

To overcome these barriers, it is crucial to develop effective interventions that target knowledge, attitudes, and beliefs related to blood donation in Saudi Arabia. Education campaigns that highlight the importance of blood donation and address misconceptions and fears can help increase the number of blood donors. Additionally, providing incentives such as free health check-ups or small gifts can motivate people to donate blood. Knowledge and barriers associated with blood donation are significant issues in Saudi Arabia. Addressing these factors through effective interventions can increase the number of blood donors and contribute to the healthcare system in Saudi Arabia. It is crucial to develop culturally sensitive and targeted interventions that address the specific needs and concerns of potential blood donors in Saudi Arabia [7].

The blood transfusion service in the Kingdom of Saudi Arabia (KSA) is essentially a hospital-based blood banking system, where blood banks are in charge of every aspect of the service, including finding donors and analyzing given blood. The majority of blood donations are made directly by patients' relatives, friends, and coworkers, but there is also an increasing number of voluntary, unpaid donors [6]. However, there are alternative options, such as the Red Crescent, local blood banks, and blood donation campaigns. This systematic review aims to comprehensively investigate the level of knowledge and attitudes towards blood donation in Saudi Arabia.

## Review

Methods

This systematic review complied with established criteria from the Preferred Reporting Items for Systematic Reviews and Meta-Analyses (PRISMA) [7].

Study design and duration

This systematic review was conducted in September 2023.

Search strategy

A thorough search was conducted across four main databases, including PubMed, Scopus, Web of Science, and Science Direct, to locate the pertinent literature. We limited our search to English and considered each database's particular needs. The studies corresponding to the following keywords were located using PubMed Medical Subject Headings (MeSH) terms: "blood donation," "blood transfusion," "knowledge," "awareness," "perception,", and "Saudi Arabia." Boolean operators "OR" and "AND" matched the required keywords. Publications with full English text, available free articles, and human trials were among the search results.

Inclusion and exclusion criteria

We considered the following criteria for inclusion in this review: study designs that investigated the level of knowledge and attitudes towards blood donation in Saudi Arabia; adult-based studies; human subject studies; English-language studies; and free, accessible articles. We excluded studies outside Saudi Arabia, studies on children, articles in other languages than English, and closed-access articles.

Data extraction

Rayyan (Qatar Computing Research Institute, Ar-Rayyan, Qatar) was used to check the output of the search technique for duplication [8]. The researchers assessed the titles' and abstract relevance by altering the combined search results with inclusion/exclusion criteria. The reviewers thoroughly scrutinized each paper that matched the inclusion criteria. The writers discussed dispute-resolution approaches. The authorized study was uploaded using a previously generated data extraction form. The authors extracted data about the study titles, authors, study year, country, participants, gender, type of population, and complications. A separate sheet was created for the risk of bias assessment.

Strategy for data synthesis

Summary tables were created using data from relevant studies to provide a qualitative interpretation of the findings and study components. After retrieving the data for the systematic review, the most efficient strategy to use the data from the included study articles was chosen.

Risk of bias assessment

The quality of the included studies was assessed using the Risk of Bias in Non-randomized Studies of Interventions (ROBINS-I) risk of bias assessment approach for non-randomized trials of treatments [9]. Confounding, participant selection for the study, classification of interventions, deviations from intended interventions, missing data, assessment of outcomes, and selection of the reported result were the seven themes evaluated.

Results

**Figure 1:**
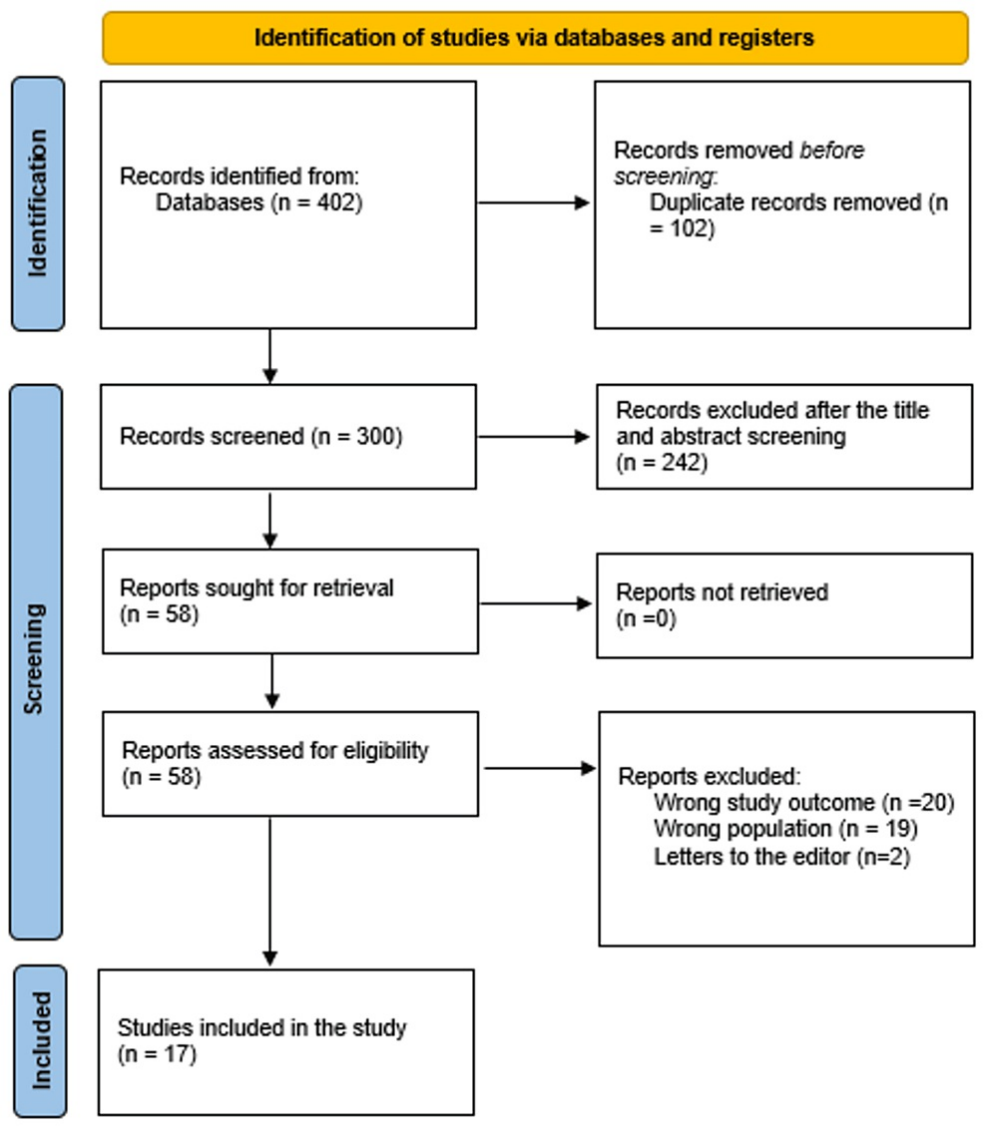
A PRISMA flowchart summarizes the study selection process. PRISMA: Preferred Reporting Items for Systematic Reviews

A total of 402 study articles resulted from the systematic search, and 102 duplicates were deleted. Title and abstract screening were conducted on 300 studies, and 242 studies were excluded. Fifty-eight reports were sought for retrieval, and no articles were retrieved. Finally, 58 studies were screened for full-text assessment; 20 were excluded for the wrong study outcomes, 19 for the wrong population type, and two articles were letters to the editors. Seventeen eligible study articles were included in this systematic review. A summary of the study selection process is presented in Figure 1.

Table 1 presents the sociodemographic characteristics of the included study articles.

**Table 1:** Study type and sociodemographic characteristics of the included participants *NM: not mentioned

Study	Type of population	Study design	City	Participants	Mean age (years)	Males (%)
Alharbi et al., 2018 [10]	Saudi nationals	Cross-sectional	Hail	717	30	442 (61.6%)
Syed et al., 2022 [11]	Saudi nationals	Cross-sectional	Riyadh	391	18-49	369 (94.4%)
Al-Drees, 2008 [12]	Saudi nationals	Cross-sectional	Riyadh	609	15-65	335 (55)
Kabrah et al., 2022 [13]	Saudi nationals	Cross-sectional	Riyadh	1506	25±2.6	221 (14.7)
Almulhim et al., 2020 [14]	Saudi nationals	Cross-sectional	Al-Khobar	1052	36.5 ± 12.63	451 (66.5%)
Mahfouz et al., 2021 [15]	University students	Cross-sectional	Jazan	468	NM	258 (55.1)
Baig et al., 2013 [16]	University students	Cross-sectional	Riyadh	326	22 ± 2.3	326 (100)
Alsalami et al., 2019 [17]	Health professionals students	Cross-sectional	Jeddah	598	21 ± 2	344 (57.5)
Albalawi et al., 2019 [18]	University students	Cross-sectional	Tabuk	161	NM	44 (27)
Felimban et al., 2019 [19]	University students	Cross-sectional	Jeddah	350	22	61 (17.4)
Alfouzan, 2014 [20]	Medical personnel	Cross-sectional	Riyadh	349	NM	161 (46.1)
Alam & El Din 2004 [21]	Saudi nationals	Cross-sectional	Sharourah	500	17-60	500 (100)
Otifi et al., 2020 [22]	Saudi nationals	Cross-sectional	Abha	844	NM	557 (66)
Abolfotouh et al., 2014 [23]	Saudi nationals	Cross-sectional	Riyadh	469	NM	356 (75.9)
Hamaki et al., 2022 [24]	University students	Cross-sectional	Al-Baha	70	21.5 ± 2.3	0
Alaskar et al., 2021 [25]	University students	Cross-sectional	Riyadh	302	18-30	0
Almutairi et al., 2018 [26]	Saudi nationals	Cross-sectional	Riyadh	500	NM	381 (76.2)

Our results included 17 studies with a total of 9,212 participants; 4,806 (52.2%) were males. All of the included articles were cross-sectional studies [10-26].

Table 2 presents the clinical characteristics of the included studies.

**Table 2:** Clinical outcomes of the included studies FAMS: Foundation for Applied Medical Sciences; KAP: knowledge, attitude, and practice

Study	Main outcomes	ROBIN-I
Alharbi et al., 2018 [10]	Saudi Arabia still has a weak understanding of blood donation in its entirety.	Moderate
Syed et al., 2022 [11]	Blood donation and its significance in society and the healthcare system are topics that the Saudi public accepts as having acceptable knowledge and favorable opinions. The participants' educational background and gender are strongly related to the participants' knowledge. Young donors were substantially connected with favorable beliefs.	Moderate
Al-Drees, 2008 [12]	65.84% of the participants were non-donors. The non-donor group added that the distance to the donation site, the difficulty with transportation, the time commitment, getting a brief break from work or the office, or taking time off from home, as well as various fears, mistrust, a lack of information, and not being approached by anyone to donate were what most discouraged them from giving blood. The current study's participants lacked enough knowledge of blood supply in blood banks, blood donation, and blood transfusion.	High
Kabrah et al., 2022 [13]	By examining the knowledge score, it was shown that 81.9% of participants knew how important it is to check a donation for infections. Regarding attitude, 74.2% of respondents strongly agreed that more people should be aware of the value of blood donation.	Moderate
Almulhim et al., 2020 [14]	The respondents had poor general knowledge and a positive attitude toward giving. Most respondents thought incentives were unnecessary but nevertheless welcomed them.	High
Mahfouz et al., 2021 [15]	Blood donation information among the students seemed to be insufficient. Additionally, blood donation is not very common. The primary reasons for not donating blood include being ineligible, fear of blood donation, and misinformation about blood donation.	High
Baig et al., 2013 [16]	Young Saudi University students' awareness of blood donation is subpar, and numerous myths are pervasive in their society.	Moderate
Alsalami et al., 2019 [17]	Academic standing and blood donation knowledge were strongly related to the practice. The college's contribution to the cause of blood donation was also noticeably absent.	Moderate
Albalawi et al., 2019 [18]	As the foundation of the community and future doctors, medical students' declining understanding of blood donation and their attitudes toward autologous blood transfusion are seen as a serious issue that requires more attention.	High
Felimban et al., 2019 [19]	The low rates of blood donors among FAMS students are not a result of a lack of understanding of the need for blood transfusions for a patient's survival and the community's general well-being. The unfavorable replies from the FAMS students serve as a foundation for upcoming awareness initiatives on blood donation that should assist in attracting donors and maintaining their motivation to donate blood regularly.	Moderate
Alfouzan, 2014 [20]	The study population's knowledge of various aspects of blood donation is insufficient. However, they often have a positive attitude about it. Males, highly educated individuals, those in the military, and those between the ages of 31 and 50 were more likely to donate blood than were younger individuals, females, those with less education, and students.	Moderate
Alam & El Din 2004 [21]	Basic information on blood donation is lacking among non-donors. When asked why they did not often donate blood, they said they could not give a reason but that they were willing to do so if asked. The Saudi population has certain misconceptions about blood donation.	High
Otifi et al., 2020 [22]	Low levels of awareness exist regarding blood donation. This study's key finding was that people were more inclined to donate blood if they had attended awareness programs.	Moderate
Abolfotouh et al., 2014 [23]	The Saudi population's perception of blood donation was less than ideal, most likely as a result of misunderstandings, a lack of awareness, and a negative attitude toward giving.	Moderate
Hamaki et al., 2022 [24]	The majority of the female students had a positive attitude towards blood donation, but only roughly one-third of them had excellent knowledge of it.	Moderate
Alaskar et al., 2021 [25]	Low levels of previous blood donation were present in the sample. This is caused, in part, by a lack of understanding of the donation procedure.	Moderate
Almutairi et al., 2018 [26]	Most Saudi individuals had good KAP scores and demonstrated a high incidence of blood donation among them, as well as a favorable attitude towards blood donation.	Moderate

Lack of knowledge, negative attitudes, and misconceptions were predominant among the Saudi public population [10, 12-14, 21-23]. Participants reported that the distance to the donation site, the difficulty with transportation, the time commitment, getting a brief break from work or the office, or taking time off from home, as well as various fears, mistrust, a lack of information, and not being approached by anyone to donate were the reasons for the negative attitudes towards blood donation. However, Saudi university students had insufficient knowledge but positive attitudes toward blood donation [15-20, 24, 25]. Young participants were more willing to donate. The primary reasons for not donating blood include being ineligible, fear of blood donation, and misinformation about blood donation.

Discussion

Millions of people depend on the practice of blood donation as a life-saving action during various emergencies and medical conditions. Due to the rise in disorders, including cancer, anemia, malaria, pregnancy-related illnesses, and auto accidents, the availability of healthy blood is urgently needed. Therefore, this systematic review studies the knowledge and availability of the Saudi population to donate blood.

The general population lacked knowledge and had negative attitudes and misconceptions toward blood donation [10, 12-14, 21-23]. Another systematic review and meta-analysis conducted by Getie et al. were consistent with our results and reported that there is a lack of sufficient information about blood donation in Ethiopia, as evidenced by the pooled prevalence of good knowledge about blood donation being 56.57% (95% CI 50.30 to 62.84) [27]. This can be because the nation lacks regular blood donation programs, adequate media attention, effective blood donation campaigns, and adequate educational opportunities.

In this study, participants reported that the distance to the donation site, the difficulty with transportation, time commitment, getting a brief break from work or the office, or taking time off from home, as well as various fears, mistrust, a lack of information, and not being approached by anyone to donate were the reasons for the negative attitudes towards blood donation. Blood transfusion facilities need to know what encourages and inhibits giving in order to keep donors [28]. Donation obstacles and motivations should be taken into account. A barrier is anything that prevents or inhibits donation (such as a fear of needles, a lack of time, or physical issues), whereas a motivator could be anything that encourages donation (such as prosocial motivations, pleasant feelings, or rewards). Their simultaneous analysis is explained by the interplay between obstacles and motivations: people choose not to give when barriers outweigh motivations in terms of number and/or intensity, and vice versa [29]. Though many reasons have additive impacts on the decision to donate, a single obstacle may completely preclude a donation effort [30]. This is where there is particular interest in researching barriers in relation to their impact on donation behavior. Despite their willingness to do so, most of the population does not donate blood due to this [31].

However, Saudi university students in this study had insufficient knowledge but positive attitudes toward blood donation [15-20, 24, 25]. Young participants were more willing to donate. The primary reasons for not donating blood include being ineligible, fear of blood donation, and misinformation about blood donation. There hasn't been much focus on the link between education and donation barriers. Studies already published link less educated donors to medical reasons [32] and a lack of knowledge about where to donate [33]. Contrarily, donors with a university education are more likely to lack time [32, 34] and fail to meet the donation requirements since they are traveling to specific nations.

The exposition stated above leads to two conclusions. Firstly, studies already conducted on the subject have a tendency to concentrate on the same hurdles, which are mostly those caused by anxiety, a lack of time, and medical conditions. Second, it is clear that the sociodemographic profile of the donor affects the likelihood that particular donation barriers will exist.

## Conclusions

The findings of this study shed light on the prevailing negative attitudes and misconceptions toward blood donation among the general Saudi population. It was observed that several factors contributed to these negative attitudes, including the distance to the donation site, transportation difficulties, time commitment, work or home responsibilities, as well as fears, mistrust, and a lack of information. Additionally, the absence of any encouragement or approach from others to donate further contributed to this trend. However, it is worth noting that university students displayed a different outlook. While they exhibited insufficient knowledge about blood donation, they maintained positive attitudes towards it. Furthermore, the study revealed that younger participants were more inclined to donate blood.

Nonetheless, there were still barriers preventing individuals from donating blood. These barriers included ineligibility, fear associated with the act of blood donation, and misinformation surrounding the process. There is a need for increased awareness and education regarding blood donation among the general Saudi population. Efforts should be made to address the identified barriers and misconceptions to promote a more positive attitude towards blood donation and ultimately increase the number of donors.
